# Pustular Acne

**Published:** 1885-12

**Authors:** Henry Wile

**Affiliations:** Lecturer on Diseases of the Skin in the Atlanta Medical College, Atlanta, Ga.


### PUSTULAR ACNE.

A CLINICAL LECTURE DELIVERED OCTOBER 31ST, BY HENRY WILE, M. D., LECTURER ON DISEASES OF THE SKIN IN THE ATLANTA MEDICAL COLLEGE, ATLANTA, GA.

Reported for THE ATLANTA MEDICAL AND SURGICAL JOURNAL.

This case, gentlemen, represents a class of cutaneous diseases that often comes to the notice of the general practitioner, and the treatment is generally regarded as unsatisfactory.

This boy, colored, nineteen years of age, exhibits a pin-head sized discrete pustular eruption scattered about on the face, especially on the cheeks and forehead. The eruption, the patient tells us, has existed for several years, and the lesions appear and disappear in successive crops. Examining the skin on the face of the patient closely, you will see that the pustules are situated around the outlets of the sebaceous glands. Furthermore, that these outlets are abnormally dilated and, in many places, filled with sebaceous plugs, known as comedones or black-heads. The surface of the skin also has an oily look, showing that the sebaceous glands are unusually active. In addition, there is still another characteristic symptom of this disease in the numerous pin-head sized scars here and there. These scars show that the process has been going on for some time, and that the pustulation is rather deeply seated, being accompanied by destruction of the corium.

You see here represented an inflammatory disease of the sebaceous glands, known as acne, and in this case it is of the pustular variety. This disease is pecular to the period of youth, generally appearing between the ages of fourteen and twenty. This is the period when maturity is being reached, and the tissues of the organism are unusually active, especially the sebaceous glands.

The diagnosis of this disease should never trouble any of you if you will bear in mind the following points which are eminently characteristic: Its occurrence during the period of youth; its chronicity; the lesions appearing in successive crops; the location, as a rule, on the face, and the lesions involving the sebaceous glands; co-existence of comedones and sebaceous glands with dilated outlets.

The treatment should be both internal and external, the former consisting in the correction of any derangements of digestion which often bear a causal relation to the disease. The diet must be carefully regulated, and all indigestible material, such as pastries, salt meats, cheese, cabbage, fresh bread, biscuit, etc., should be interdicted. The form of dyspepsia commonly encountered in connection with acne is that due to an excess of acid secretion in the gastric juice. In this case the following may be used with benefit:

| | |
|---|---|
| ℞—Sodii bicarbonatis . . . . . . . . . . | ʒ ii |
| Potassii acetatis . . . . . . . . . . . . | ℥ s s |
| Aquæ menthæ piperitae . . . . . . . . | f℥ i |
| Infus. quassæ . . . . q.s . ad. . . | f℥ iii |

M.—

Sig.—One teaspoonful in a wineglass of water one-half hour after meals.

Where constipation exists the following pill may be ordered:

| | |
|---|---|
| ℞.—Aloes . . . . . . . . . . . . . . | gr. xl. |
| Ext. belladonnæ . . . . . . , . . . . | gr. iii. |
| Ext. nucis vomicæ . . . . . . . . . . | gr. viii. |

M.—Ft. mass.

Div. in pill No. xv.

Sig.—One at bed time.

The external treatment of acne depends upon the character of the process, whether acute or chronic, upon the extent of the lesions and the general condition of the skin. If acute, and where there is much irritation, a mild lotion or ointment is indicated. A lotion such as—

℞—Pulveris calaminæ.

Zinci oxidi . . . . . . . . a.a . . . ʒ i

Glycerini . . . . . . . . . . . . . f℥ ss.

Aquæ rosæ . . . . . . . . . . . . . f℥ iv

M.—Ft. lotio.

Sig.—Apply for five minutes, morning and evening.

When the process is chronic, as is generally the case, and such as you see here in this patient, more stimulating remedies are called for—such as the preparations of sulphur or the mercurials. Strong sulphur ointments, such as precipitated sulphur, two to four drachms to the ounce of vaseline, are specially valuable.

In this case, gentlemen, there is, as I intimated, need of stimulation, and as there is a more or less seborrhœic condition co-existing, I will order the following lotion, which I consider a most happy combination of remedies. The lotion was originally proposed about two years ago as a remedy for lupus erythematosus, by Prof. L. A. Duhring, of Philadelphia, but has since been used with success in the treatment of acne. It is composed as follows :

℞.—Zinci sulphatis.

Potassæ sulphuretæ . . . . . . a. a. . ʒ i.

Alcohol . . . . . . . . . . . . . . fʒ iii.

Etheris . . . . . . . . . . . . . . fʒ iii

Aquæ Rosæ . . . . . . . . . . . . f℥ iv.

M.—Ft. lotio.

Sig.—Apply twice daily, five to ten minutes at a time.

The patient will also be ordered to bathe the face with hot water before making the application. Before prescribing any medicine internally, we will direct him to take a tablespoonful of Rochelle salts in a tumbler of water before breakfast for two consecutive mornings. As to prognosis in cases of acne, you must tell the patient, at the very outset, that patience, perseverance and a strict adherence to the methods of treatment are requisite. A cure under such conditions can, as a rule, be promised.

